# Artificial Intelligence Image Recognition System for Preventing Wrong-Site Upper Limb Surgery

**DOI:** 10.3390/diagnostics13243667

**Published:** 2023-12-14

**Authors:** Yi-Chao Wu, Chao-Yun Chang, Yu-Tse Huang, Sung-Yuan Chen, Cheng-Hsuan Chen, Hsuan-Kai Kao

**Affiliations:** 1 Department of Electronic Engineering, National Yunlin University of Science and Technology, Yunlin 950359, Taiwan; alanwu@yuntech.edu.tw; 2 Interdisciplinary Program of Green and Information Technology, National Taitung University, Taitung 950359, Taiwan; 10822110@gm.nttu.edu.tw (C.-Y.C.); 10822172@gm.nttu.edu.tw (Y.-T.H.); 10822120@gm.nttu.edu.tw (S.-Y.C.); 3 Department of Electrical Engineering, National Central University, Taoyuan 320317, Taiwan; 048853@mail.fju.edu.tw; 4 Department of Electrical Engineering, Fu Jen Catholic University, New Taipei City 242062, Taiwan; 5 Department of Orthopedic Surgery, Chang Gung Memorial Hospital at Linkou, Taoyuan 333423, Taiwan; 6 Bone and Joint Research Center, Chang Gung Memorial Hospital at Linkou, Taoyuan 333423, Taiwan; 7 College of Medicine, Chang Gung University, Taoyuan 333423, Taiwan

**Keywords:** intelligent image recognition, wrong-site left and right upper limb surgery, accuracy rate, recall rate, IRB

## Abstract

Our image recognition system employs a deep learning model to differentiate between the left and right upper limbs in images, allowing doctors to determine the correct surgical position. From the experimental results, it was found that the precision rate and the recall rate of the intelligent image recognition system for preventing wrong-site upper limb surgery proposed in this paper could reach 98% and 93%, respectively. The results proved that our Artificial Intelligence Image Recognition System (AIIRS) could indeed assist orthopedic surgeons in preventing the occurrence of wrong-site left and right upper limb surgery. At the same time, in future, we will apply for an IRB based on our prototype experimental results and we will conduct the second phase of human trials. The results of this research paper are of great benefit and research value to upper limb orthopedic surgery.

## 1. Introduction

Even with advances in medical technology, medical errors due to human error are still bound to occur. According to the research report To Err is Human: Building a Safer Health System published by the U.S. National Institute of Medicine in 2000, the rate of medical errors caused by human negligence is as high as 2.9%, and over 50% of medical errors are preventable. Hence, how to avoid medical errors caused by human negligence has always been an important research topic in the medical field. Among all medical procedures, surgery has always been the riskiest. Among the types of surgical medical negligence, incorrect identification of surgical site, defined as making an incision on the incorrect anatomical region, is among the most common. In orthopedic surgery, the wrong site of surgery includes the identification of the wrong operation position and the wrong operation site. Taking the wrong incision position as an example, assume that the patient’s original incision was intended for the right limb, but after anesthesia, due to human error, the incision was performed on the left limb. Alternatively, the surgery was intended to be performed on the right elbow, but after anesthesia, the surgery was performed on the right wrist. Such operations are often simple operations, but performing surgery on the wrong surgical site causes more serious injuries to the patient; moreover, the medical team and the hospital could face significant compensation claims, pressure from public opinion, and even legal proceedings [1]. Therefore, how to avoid medical errors caused by human negligence remains a primary research topic in the medical field.

**Figure 1:**
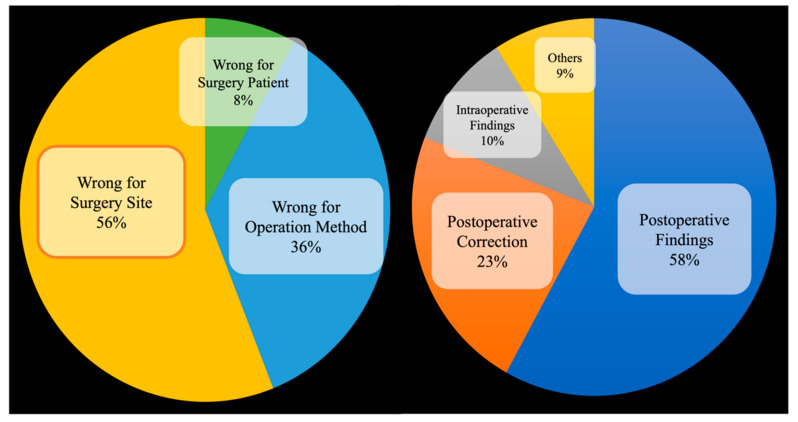
Categories of surgical site errors [3].

Among all medical errors, surgical medical errors often cause significant injuries and losses to patients and medical institutions, since surgery is the most prevalent medical action. Medical negligence in surgery is divided into several categories. Among them, in the 2019 annual report of the Taiwan Patient Safety Reporting System [2], it was pointed out that the incidence of surgical site errors reached second place among the most common types of surgical error events. How to avoid surgical site errors is an important research issue in the field of medical malpractice. As shown in Figure 1, among all surgical specialties, as many as 56% of errors in orthopedic surgery are surgical site errors, and because orthopedic surgery pertains to skeletal and muscular injuries of the extremities, only 5.4% can be corrected before surgery [3].

In view of this, after discussions with the supervisor and the cooperating orthopedic surgeon, this project will first take the example of upper limb orthopedic surgery as the main surgical site to propose a smart image recognition system that can prevent identification errors between the left and right upper limb in orthopedic surgery and assist orthopedic surgeons in preventing upper limb surgery left–right misalignment errors.

**Figure 2:**
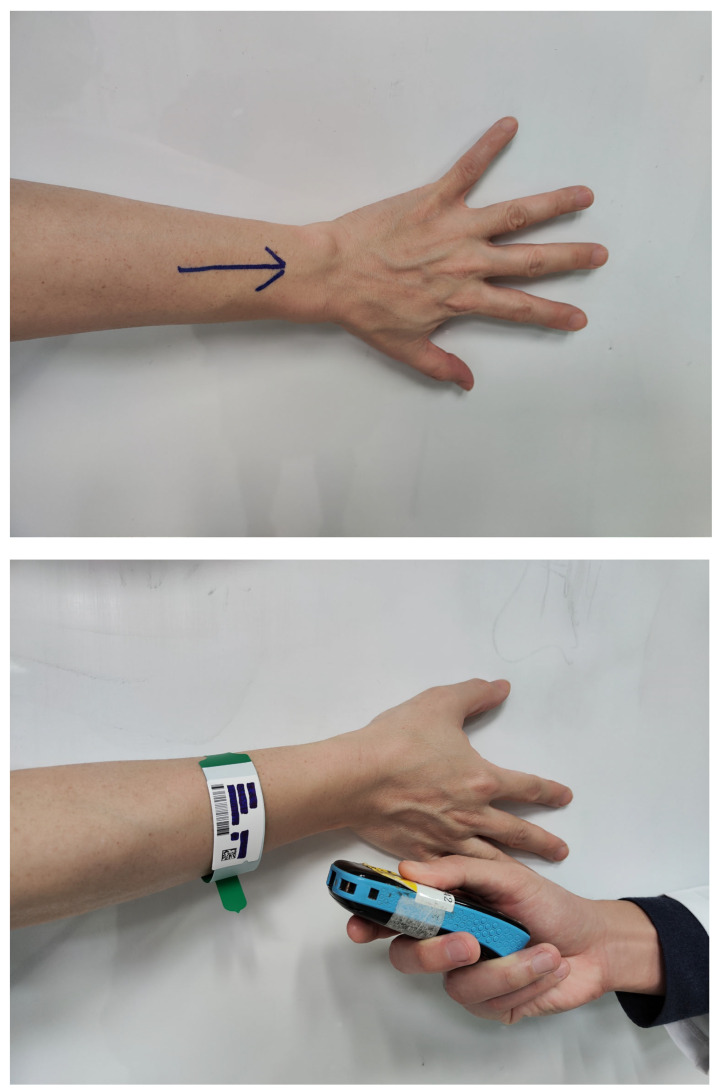
Marking and barcode scanning.

Contemporary measures aimed at preventing erroneous upper limb surgeries often rely on marking or barcode scanning methodologies, as shown in Figure 2. Nonetheless, the susceptibility to human error and external influences remains a notable drawback. In contrast, the Artificial Intelligence Image Recognition System, AIIRS, hinging on a YoloV4-based deep learning model, effectively discerns left and right upper limbs within medical imagery, eliminating the reliance on external markings or scanning devices. Notably, the AIIRS showcases a commendable level of accuracy and recall rates in image recognition.

In a systemic review on wrong surgical sites published by Susanne Hempel in JAMA Surgery in 2015, it was pointed out that the incidence of surgical site errors is about 1 in 100,000. The major reason for the occurrence of these mistakes was poor communication between medical personnel. Poor communication included miscommunications among staff, missing information that should have been available to operating room staff, surgical team members not speaking up when they noticed that a procedure targeted the wrong side, and surgeons ignoring surgical team members when laterality was questioned [3]. Medical teams must communicate fully and not be afraid to ask questions. Chief surgeons must also respect everyone’s opinions to avoid medical negligence in terms of wrong surgical site.

In a study published by Mark A Palumbo in 2013, it was pointed out that it is not only the limbs that can be operated on in the wrong place—wrong-site spinal surgery often occurs. If the wrong site is prescribed, it is devastating for both the patient and the doctor. Hence, it was suggested that strict regulations should be put in place to prevent wrong-site surgery. It is also necessary to develop a customized process (patient-specific protocol) to avoid wrong-site surgery [4].

Omid Moshtaghi pointed out that, although California introduced a universal surgical safety protocol in 2004 to ensure the safety of patients during surgery, wrong-site surgery still continues to occur. In terms of disciplines, wrong-site surgery was the most common in orthopedics, accounting for 35% of cases [5].

The authors pointed out that orthopedics is the department with the most frequent surgical site errors. The most common causes included a breakdown in communication, time pressure, emergency procedures, multiple procedures on the same patient by different surgeons, and obesity. Careful pre-surgery checks were recommended to determine effective solutions [6].

Currently, during orthopedic surgery, a mark is drawn on the surgical site to remind the surgeon where to draw the knife before the skin is disinfected and draped. Currently, the site of orthopedic surgery is mostly marked by using a colored pen to draw a mark on the relevant body part, as shown in Figure 2. Some hospitals adopt the barcode scanning method and use a barcode machine to confirm whether the position is correct, as shown in Figure 2. However, regardless of marking or barcode, wrong-site surgery may still occur due to the following factors: initiating or completing surgery under unusual time pressure, incorrect instrument setting or handling in the operating room, referral of patient to another physician, physicians from multiple disciplines participating in the operation, and unusual physical features requiring special positioning.

Based on the above literature review, traditional methods should still currently be used to prevent wrong-site surgery. These include strengthening communication, identifying patients correctly, marking the surgical site, time-out before surgery, and using check lists. Stricter inspections and checks are needed to prevent the occurrence of surgical site errors.

Among intelligent image recognition technologies, deep learning image recognition is generally used. This type of image recognition method mainly uses a large number of training data sets to find the characteristics of the image to be recognized. However, the current deep learning image recognition technology cannot be applied to the intelligent image recognition system for left and right upper limb orthopedic surgery discussed in this study, since the left–right symmetry of the human body is the golden ratio. If there are no special marks, birthmarks, or scars on the left or right upper limbs, the image features of the left and right upper limbs will be the same.

Hence, this paper aimed to develop an upper and lower limb surgical site identification system based on deep learning. In the first stage, a dummy was used as the test object. After the deep learning model training is mature, IRB approval will be sought. In the second stage, human trials will be conducted. 

After discussing these issues with an orthopedic surgeon (corresponding author), it was concluded that it is absolutely necessary to develop an orthopedic upper limb intelligent image recognition system to replace the marking and barcode machine scanning methods before surgical disinfection and draping. Orthopedic surgery accounts for 41% of wrong-site surgery overall, putting it in first rank. Among wrong-site errors, left and right position errors are the most commonver, most research related to upper limb surgery focuses on rehabilitation, lymph, and the nerves [7,8,9,10,11,12,13,14,15,16,17,18,19,20,21,22,23,24,25,26,27,28,29]. No research focused on using image recognition to distinguish between the left and right upper limbs to prevent wrong-site surgery was found. Developing a new type of intelligent image recognition system to help surgeons distinguish the left and right upper limbs to prevent wrong-site surgery during orthopedic upper limb surgery would bring considerable benefits and research value.

This paper adopted YoloV4 as the main deep learning model. The analysis of CNN-based architecture included CSPDarknet53, SPP (Spatial Pyramid Pooling), and PANet (Path Aggregation Network). In addition, Yolo head and mish activation were also included. CSPDarknet53 is an improved version of Darknet. It is designed to improve information fluidity and accelerate training convergence and detection performance. SPP allows the network to process different sizes of input images to improve the model’s detection of targets on different scales. PANet was used to fuse features on different scales to improve the accuracy of target detection, especially for small targets. Yolo head is responsible for predicting the location, category, and confidence of the object. In addition, anchor boxes were used to predict the location of objects. Mish activation is a non-linear activation function to improve the learning ability of the model. The main contribution of this paper is the combination of existing deep learning models to achieve intelligent image recognition of the left and right upper limbs for orthopedic surgery through different new data training sets and test training sets and the coordination of model parameters with the goal of preventing wrong-site upper limb surgery.

This paper endeavors to address the critical issue of mitigating erroneous upper limb surgeries through the implementation of an Artificial Intelligence Image Recognition System (AIIRS). The pivotal research query revolves around the system’s precision and consistency in discerning between the left and right upper limbs in medical imagery, thereby aiding orthopedic surgeons in averting potential procedural errors. The seminal contribution of this paper resides in the development and evaluation of the AIIRS. It is a groundbreaking Artificial Intelligence Image Recognition System designed specifically to counter the issue of erroneous upper limb surgeries. To the best of our knowledge, this study represents a pioneering application of deep learning image recognition in the context of mitigating erroneous upper limb surgeries, showcasing its feasibility and efficacy within the confines of a comprehensive pilot study.

To comply with academic theory and human research ethics, laboratory students were used as dummy orthopedic surgery patients. Their images were used to establish a training data set and an initial test data set. After the prototype of the deep learning model is completed and trained, IRB approval will be applied for and the second phase of human trials will take place.

The subsequent segments of this paper comprise an in-depth exposition of the materials and methods adopted (Section 2), a comprehensive report on the experimental findings and analyses (Section 3), a critical discussion on the study’s implications and limitations (Section 4), and a concluding segment that not only synthesizes the findings but also offers recommendations for future research directions.

## 2. Materials and Methods

Current position marking for orthopedic surgery is mainly based on marking or barcode scanning. However, these methods may still generate surgical site errors due to time pressure, unfamiliar instrument setup or handling, participation of multiple surgeons, patients being referred to another physician, and other factors. Hence, preventing wrong-site surgery is absolutely the top priority of orthopedic surgery.

In this paper, the corresponding author, Dr. Hsuan-Kai Kao, was also an orthopedic surgeon in the department of Orthopedic Surgery, Chang Gung Memorial Hospital, Linkou, Taiwan. According to discussions with Dr. Kao, the marking and barcode scanning methods are still used in the hospital without using any artificial intelligence method for orthopedic surgery. In addition, wrong-site upper limb surgery often occurs in the department. Hence, this paper proposed an Artificial Intelligence Image Recognition System, AIIRS, combined with a deep learning neural network to assist orthopedic surgeons and prevent wrong-site upper limb surgery. In an internal survey of the members of the Orthopedic Medical Association of the Republic of China, it was shown that as many as 56% of errors in orthopedic surgery are left–right errors, and only 5.4% could be corrected before the operation.

As assessed through actual visits in the hospital and discussions with an orthopedic surgeon, who is also the corresponding author of this paper, the medical unit currently still uses marking or barcode scanning to recognize the left and right positions of the upper limbs for orthopedic surgeries, without any AI image recognition. Although many AI medical image recognition methods have been proposed, none of them was applied to recognize the left and right positions in upper limb orthopedic surgery. Therefore, the AIIRS proposed in this paper could help avoid left and right identification errors in upper limb orthopedic surgery. Therefore, the AIIRS proposed in this paper for left–right recognition during upper limb orthopedic surgery could bring considerable benefits and research value.

This paper aimed to combine an existing deep learning model based on YoloV4 with an artificial intelligence image recognition system for preventing wrong-site upper limb surgery. The architecture included the backbone, neck, and head. The backbone used CNN architecture, such as CSPDarknet53. The neck used methods of integrating various scales, such as SPP and PANet. The head used the same bounding box methods and loss function as the head of YoloV3. CSPDarknet53 is based on CSPNet (Cross-Stage Partial Network). CSPNet contains 5 CSP (Cross-Stage Partial) modules. In CSPNet, some input will be split for convolution. After the convolution is completed, it will be concatenate with prior input convolution, and then output to the input of the next layer. The output of the last layer will be concatenate with the part of the input that was not processed repeatedly. CSPNet could thus effectively alleviate the vanishing gradient problem and deepen the number of layers in the network. A Spatial Pyramid Pooling (SPP) layer increased the receptive field of the network. The maxpool method of kernel size *K* = {1×1, 5×5, 9×9, 13×13} was used. Finally, feature maps of different scales were operated with concatenation. In PANet, two feature maps are combined with a concatenate operation. The head used was the same as the head of YoloV3. No bilinear pooling with poisoning detection module was used, since it was assumed that no poisoning attacks existed in YoloV4 [30].

Our literature review showed that artificial intelligence (AI) systems are more and more important in orthopedic surgery [31]. It also summarized the current state of machine learning in the field of orthopedic surgery. Ref. [32] showed that machine learning could be used in orthopedic surgery with good results. The utility of artificial intelligence and machine learning in orthopedic surgery continues to grow and expand. In [33], it was shown that artificial intelligence and machine learning applications could help expand orthopedic surgery field. However, this research focused on lower extremity arthroplasty. None of these studies addressed the prevention of wrong-site upper limb surgery. Therefore, the combination of an existing deep learning neural network model, such as YoloV4, with the coordination of model parameters through different new training data sets and test data sets to achieve intelligent image recognition of the left and right limbs for upper limb orthopedic surgery, as proposed in this paper, is still needed.

In our neuro-heuristic analysis model, we focused on confidence score, *Accuracy*, and *Recall*. The performance evaluation of class confidence scores is shown in the following experimental results. *Accuracy* was defined as (1) a true positive (*TP*), an actual positive sample which is predicted to be a positive sample; a false positive (*FP*), an actual negative sample which is predicted to be a positive sample; a false negative (*FN*), an actual positive sample which is predicted to be a negative sample; and true negative (*TN*), an actual negative sample which is predicted to be a negative sample, respectively. *Recall* was defined as (2).

**Figure 3:**
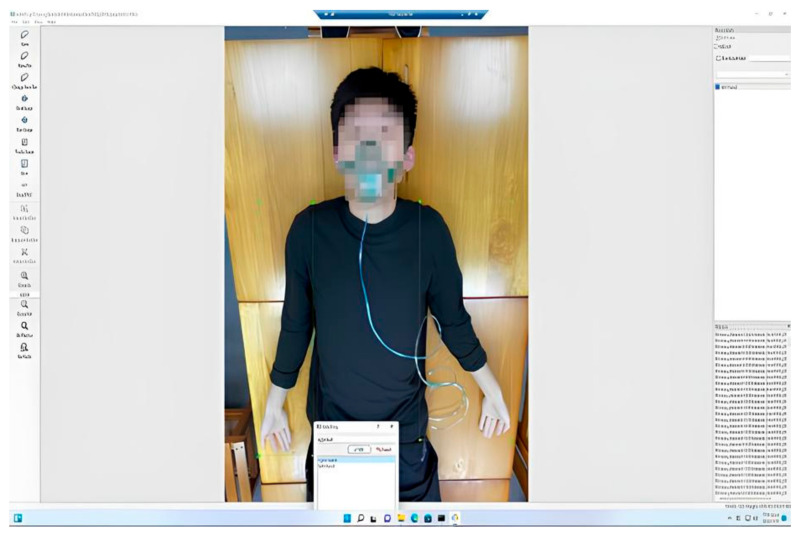
Labeling using the LabelImg software.

Among existing deep learning neural network models, Yolo was proven to be used in most image recognition applications. Hence, the neural network model in this paper adopts the most commonly used YoloV4 model. Since CSPNet in YoloV4 is based on the DarknNet53 neural network, and DarkNet53 is developed based on ResNet, two different models, namely ResNet50 and YoloV4, were used for simultaneous training. In the data set, the total number of photos was 810. Among these data, 122 photos were generated by the data generation method. The ratio of the training set to the test set is 9:1. We utilized the LabelImg software version 1.8.6 (MIT License, U.S.A.) for image-labeling purposes. The photos were labeled one by one for the training range, and divided into two parts for right-hand and left-hand training, as shown in Figure 3.

**Figure 4:**
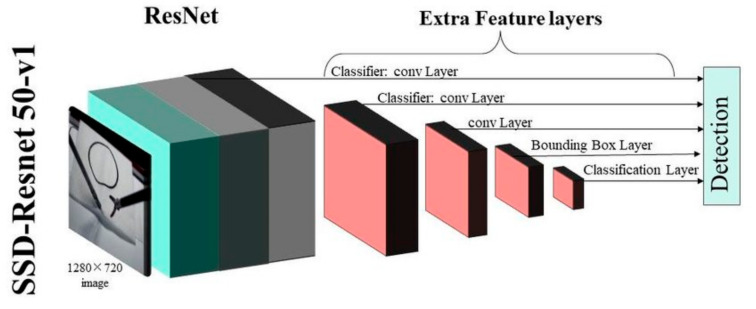
ResNet50 model [34].

**Figure 5:**
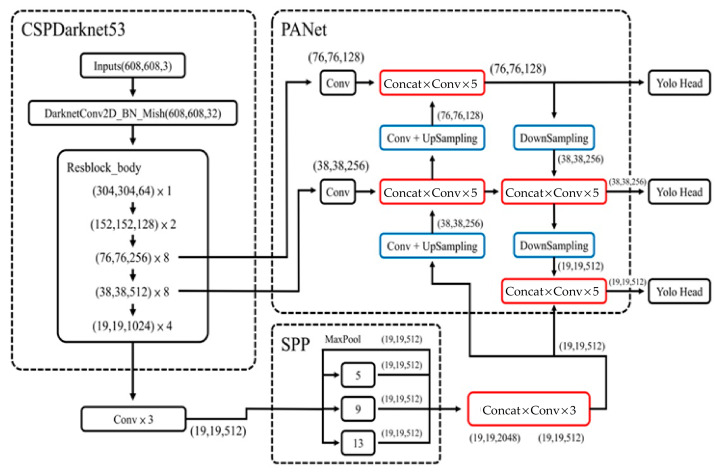
YoloV4 model [35].

In ResNet50, the marked data set was classified into a training set and a test set in two folders. The two folders were converted into.record files to ensure that the model training process could process the image files with adding a pre-training model. This model was related to the final training results. If this pre-training model was replaced, it could be transformed into other models. ResNet50 is shown in Figure 4. The pipeline.config parameter of ResNet50 is listed in Table 1 [34]. The YoloV4 parameters are listed in Table 2. The YoloV4 model is shown in Figure 5. We used the mish function as the activation function and the CIOU function as the loss function in YoloV4 [35].

In the training process, num_classed will affect the number of recognized categories, as listed in Table 1. Since there are two categories, left-hand and right-hand, in this paper, num_classed was set to 2. batch_size represents the number of input images in each iteration. As batch_size increases, the training time increases. In this paper, batch_size adopted a default value of 8. fine_tune_checkpoint_type is the pretrained model. Here, it adopted the default model of detection [34]. In Table 2, batch is defined as the batch size. Batch adopted a default value as 64 for YoloV4. max_batches is 2000 times the number of classes. If it is less than 6000, it will be set to 6000. As max_batches increases, the training time increases. The steps are 80% and 90% of 2000 times the number of classes. Since the number of classes was set to 2 in this paper, the steps were calculated as 3200 and 3600, respectively. By reducing the number of steps, the learning rate decreases during this training process. Training could be more stable with better convergence. Width and height are defined as the resized input image, 416 and 416 as default. Since there are two categories, left-hand and right-hand, in this paper, the number of classes was set to 2. Filters can be calculated as (classes+5)×3. Hence, the value of filters was set to 21. The default activation function of YoloV4 is mish. CIOU was used as the loss function in YoloV4 to calculate the difference between the predictions and the actual data [35].

In the data used, to ensure the number of training data is adequate, some data were generated by the data generation method. The number of training data was 810. For the data set, the number of photos was 680. The photos, at different angles, were taken of 15 students recruited from the laboratory to voluntarily participate. To increase the data training set, 130 photos were generated by rotating, enlarging, and decreasing some original photos. Hence, a total of 810 photos served as the training data set in this paper. The labeling method used in this paper is the LabelImg software; the xml extension format was selected for labeling. The photos were labeled one by one for the training range and divided into two different labels, right-hand and left-hand, as shown in Figure 3.

In order to enrich the training content, 729 photos were used for training and 81 photos were used for testing. The ratio of the test set to the training set was 1:9. In the pre-processing of data, each training datum was labeled one by one using the LabelImg software. The training data set was divided into right-hand data and left-hand data. In the model training program file, the paths to the training set and validation set were set first. The test set was separately distinguished and was not added to the training. The classification method was to randomly select 81 photos from the 810 photos in the data set as the test set. Training was an independent process. The test results were a separate process.

Since the AIIRS could recognize the left and right upper limbs, the AIIRS could reduce the risk of placing surgical tools and implants in the wrong position during surgery. It also could reduce human errors by medical staff. Therefore, the advantages of the AIIRS include surgical accuracy improvement and human error reduction. Although the AIIRS could accurately recognize the left and right upper limbs for orthopedic surgery, we could not use real patient data for training and testing due to privacy and security issues. Therefore, it is not certain whether the AIIRS could be applied to clinical left and right limb identification in upper limb orthopedic surgery. This is the main disadvantage of the AIIRS. In order to overcome this disadvantage, IRB approval will actively be applied for in the future to use real patient data for training and testing so that the model can be suitable for clinical left and right limb recognition for the purposes of upper limb orthopedic surgery.

To comply with academic theory and human research ethics, laboratory students were used as dummy orthopedic surgery patients. Their images were used to establish a training data set and an initial test data set. After the prototype of the deep learning model is completed and trained, IRB approval will be applied for. Then, the second phase of human trials will be conducted with the goal of achieving smart medical care through industry–university cooperation.

## 3. Results

Since this paper is a pilot study, the experiments were executed by our lab members, not real patients, to avoid seeking ethical permission. In addition, it was assumed that the lighting in the operating room is sufficient, since image recognition for left and right upper limbs would be executed before surgery.

At the small model evaluation stage, many indicators may cause confusion for performance evaluation. This paper selected two indicators, *Accuracy* and *Recall*, to measure the overall performance of our model to simplify the decision-making process. This paper focused on *Accuracy* and *Recall* since the proportion of correct classifications and correct samples in the overall model needed to be judged. Hence, *Accuracy* and *Recall* are the main performance metrics in our experimental results. *Accuracy* was defined as (1) a true positive (*TP*), an actual positive sample which is predicted to be a positive sample; a false positive (*FP*), an actual negative sample which is predicted to be a positive sample; a false negative (*FN*), an actual positive sample which is predicted to be a negative sample; and true negative (*TN*), an actual negative sample which is predicted to be a negative sample, respectively. *Recall* was defined as (2).

(1) $$Accuracy=\frac{TP+TN}{TP+FP+TN+FN}$$

(2) $$Recall=\frac{TP}{TP+FN}$$

**Figure 6:**
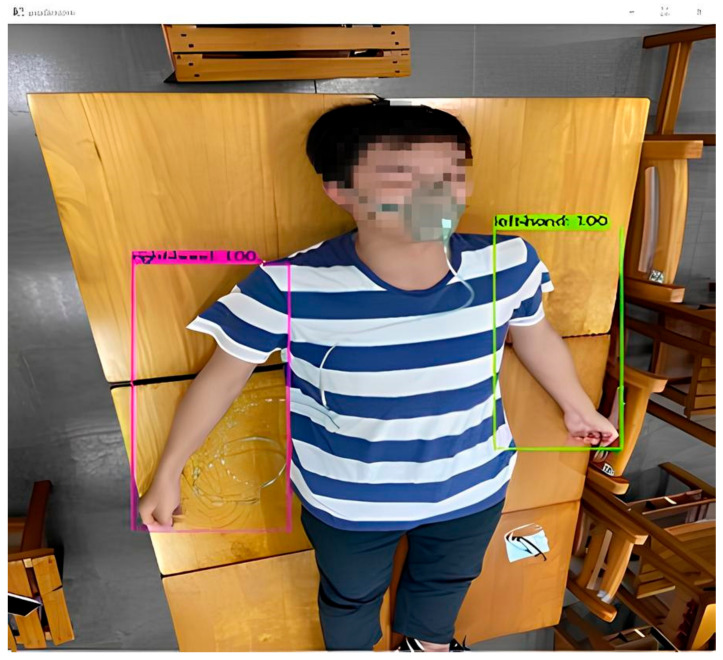

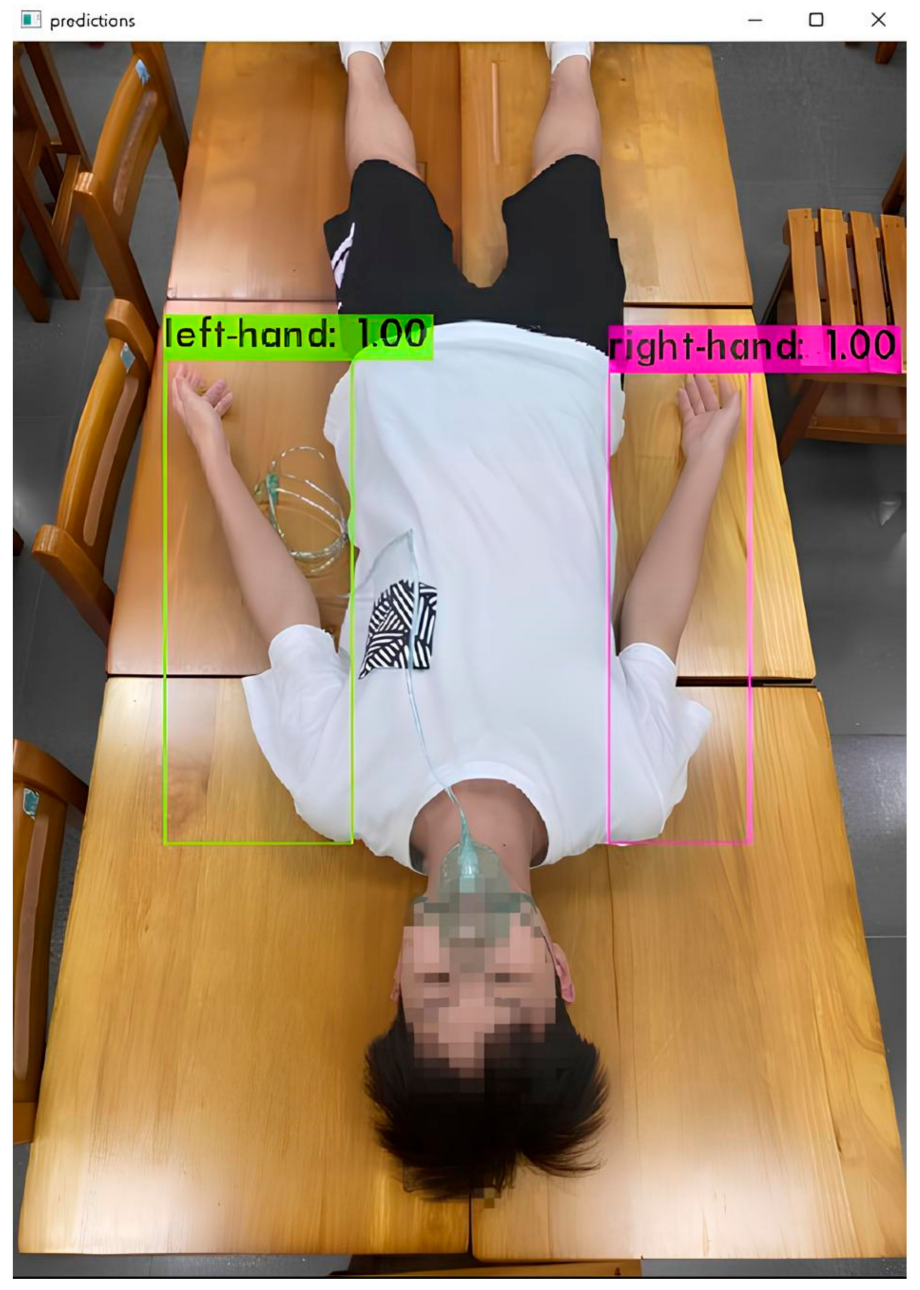
Example with *Conf_threshold* larger than 0.9.

The confidence value (*Conf_threshold*) close to the recognition frame was defined as the threshold value of object frame recognition and could be adjusted. It was also an important parameter in our proposal. For example, if the confidence value is set to 0.9, only the object frame in which *Conf_threshold* of object frame recognition is larger than 0.9 could be shown on the screen, as shown in Figure 6.

When the size of the input image is the same, a lower *Conf_threshold* will lead to an *FP*, resulting in a decrease of the overall accuracy. In order to determine the optimal *Conf_threshold*, the *Conf_threshold* was increased by 0.1 each time to calculate *Accuracy* and *Recall* with input sizes of 608×608, 512×512, and 416×416, respectively.

**Figure 7:**
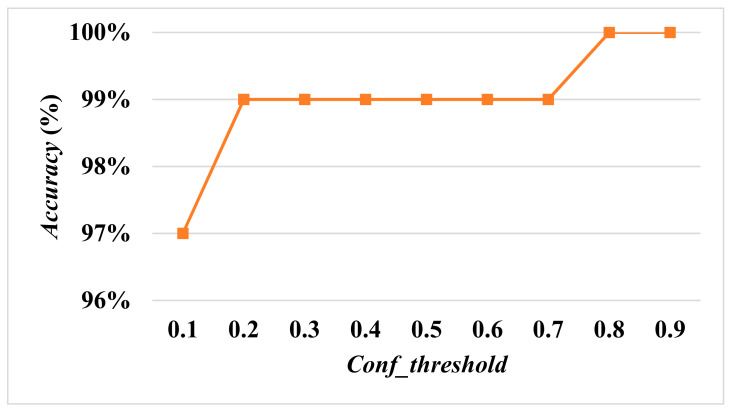
Accuracy with input size 608×608.

**Figure 8:**
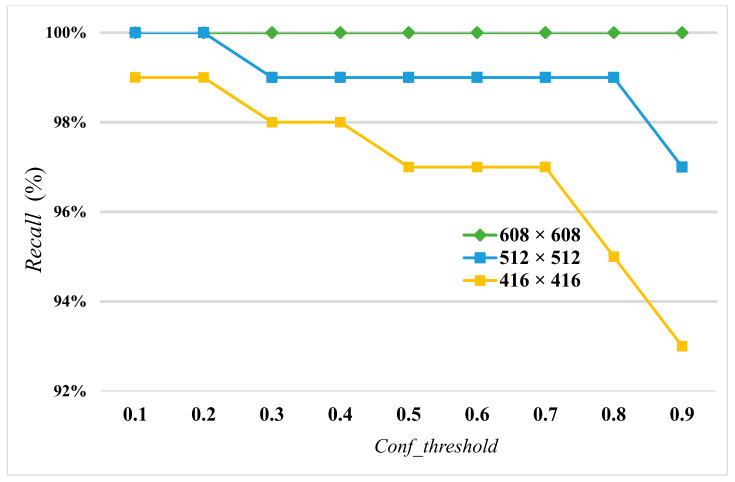
Recall with input sizes 608×608, 512×512, and 416×416.

**Figure 9:**
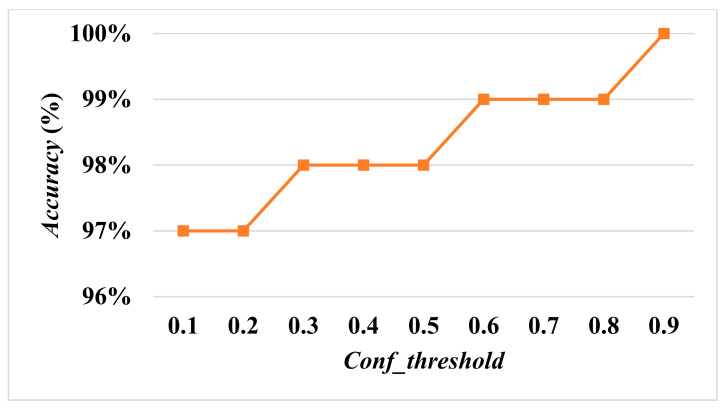
Accuracy with input size 512×512.

**Figure 10:**
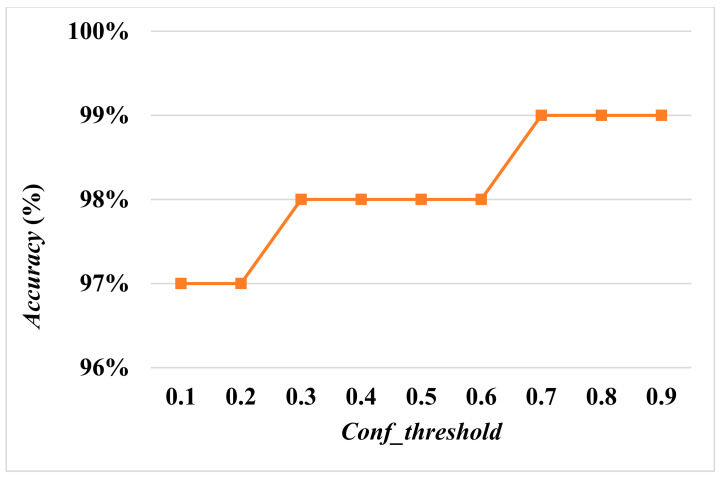
Accuracy with input size 416×416.

**Figure 11:**
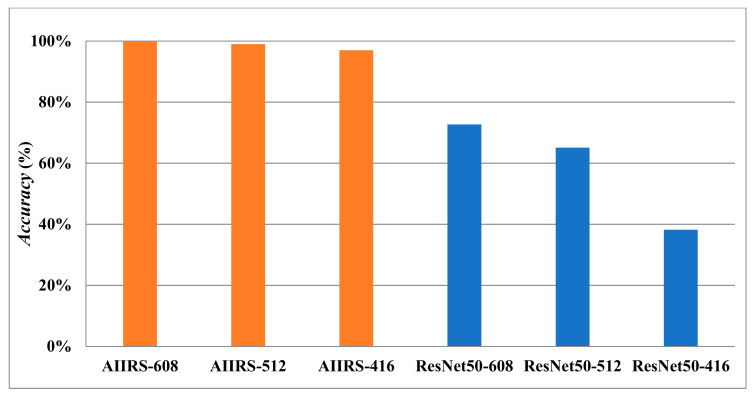
*Accuracy* under AIIRS and ResNet50 with *Conf_threshold* of 0.9 (the orange color represents AIIRS and the blue color represents ResNet50).

**Figure 12:**
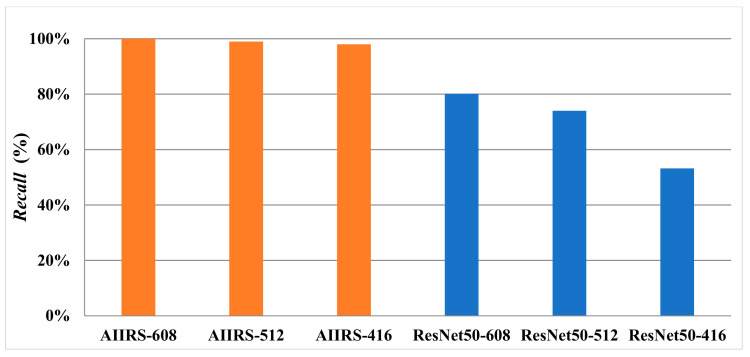
*Recall* under AIIRS and ResNet50 with *Conf_threshold* of 0.9 (the orange color represents AIIRS and the blue color represents ResNet50).

Figure 7 and Figure 8 show *Accuracy* and *Recall* with an input size of 608×608 and *Conf_threshold* increased by 0.1 each time. Figure 8 and Figure 9 show *Accuracy* and *Recall* with an input size of 512×512 and *Conf_threshold* increased by 0.1 each time. Figure 8 and Figure 10 show *Accuracy* and *Recall* with an input size of 416×416 and *Conf_threshold* increased by 0.1 each time. The experimental results in Figure 7, Figure 8, Figure 9, Figure 10, Figure 11 and Figure 12 show that the optimal *Conf_threshold* is 0.9.

The following experimental results are based on the comparison of *Accuracy* and *Recall* while *Conf_threshold* is 0.9 and input size is 608×608, as shown in Figure 7 and Figure 8, since the AIIRS had optimal *Accuracy* and *Recall* with a *Conf_threshold* of 0.9 and an input size of 608×608. AIIRS-608 is defined as the AIIRS with an input size of 608×608 and others follow in the same way. Hence, the optimal value of *Conf_threshold* was set to 0.9 for AIIRS and ResNet50, where ResNet50-608 is defined as ResNet50 with an input size of 608×608 and others follow in the same way.

Figure 11 shows that *Accuracy* with an input size of 608×608 in both the AIIRS and ResNet50 was higher than *Accuracy* with other input sizes while *Conf_threshold* was set to 0.9. It also shows that *Accuracy* in the AIIRS was higher than *Accuracy* in ResNet50. In the same way, *Recall* with an input size of 608×608 in both the AIIRS and ResNet50 was higher than *Recall* with other input sizes, as seen in Figure 12, while *Conf_threshold* was set to 0.9. Figure 12 also shows that *Recall* in the AIIRS was higher than *Accuracy* in ResNet50.

## 4. Discussion

Given that *Conf_threshold* has an impact on performance metrics such as *Accuracy* and *Recall*, our primary objective in the experimental results was to ascertain the optimal value of *Conf_threshold*. Figure 7, Figure 8, Figure 9 and Figure 10 show that the optimal *Conf_threshold* for the AIIRS was 0.9. Figure 7, Figure 8, Figure 9 and Figure 10 also show that the optimal input size for the AIIRS was 608×608. Figure 11 and Figure 12 show that *Accuracy* and *Recall* in the AIIRS were higher than *Accuracy* and *Recall*, in ResNet50. Figure 11 and Figure 12 also show that the optimal input size for ResNet50 was 608×608. Figure 8 shows that *Recall* with different input sizes (608×608, 512×512, and 416×416) was all the same. *Conf_threshold* only affected false positives (*FPs*) and true negatives (*TNs*).

To evaluate the success of the classification problem and the location of errors, in addition to *Accuracy* and *Recall*, a *confusion matrix* of randomly selected experimental results of 81 test images was created, as listed in Table 3. It proved that the AIIRS could indeed achieve left and right position recognition for upper limb orthopedic surgery.

The experimental results prove that our designed intelligent image recognition system based on YoloV4 could be applied for the prevention of wrong-site upper limb surgery. Based on the pilot experimental results, we will apply for an IRB for our artificial intelligence image recognition system (AIIRS) for the prevention of wrong-site upper limb surgery. Hence, in future, our experimental results will be closer to perfection and consistent with actual left and right upper limb position identification for orthopedic surgery.

## 5. Conclusions

Among the different types of surgical medical negligence, wrong-site surgery reached second place among the most common types of surgical error events. Orthopedic surgery is where surgical errors most commonly occur. Of these, as many as 56% are left–right errors, and only 5.4% could be corrected before the operation. Since surgical site errors are often caused by human negligence, 50% of them could be prevented. Therefore, preventing surgical site errors is a top priority in orthopedic surgery.

However, the current methods of preventing wrong site selection in upper limb orthopedic surgery are mainly marking or barcode scanning. However, the above methods are still prone to errors due to many external factors. Therefore, this study, in cooperation with orthopedic surgeons at our hospital, integrates medical and artificial intelligence technologies and develops an intelligent image recognition system for left and right side recognition during upper limb orthopedic surgery to replace the above-mentioned marking and barcode machine scanning methods.

Through image recognition of the upper limbs and machine learning technology, the system we develop can judge whether the left upper limb in the image is the left or the right upper limb. Then, doctors can obtain the correct surgical position to help orthopedic surgeons prevent the occurrence of wrong-site upper limb surgery. It is believed that the results of this project will be of considerable benefit and research value for upper limb orthopedic surgery.

To apply our research results to clinical treatment at the hospital, laboratory students were used as simulated patients in the prototype stage to complete the deep learning model. For the second phase of human trials, we will apply for an IRB. This way, the model can be actually applied to clinical medical treatment in hospitals and help to achieve smart medical treatment.

In our artificial intelligence image recognition system (AIIRS), brightness, shooting angle, and image resolution were set in advance. In addition, it was assumed that the patients would not be covered by any objects. However, these potential limitations or challenges to implementing the image recognition system may occur in real-world clinical settings. Hence, in the future, the AIIRS will be improved in terms of brightness, shooting angle, image resolution, and objects covering the patient to address concerns about its practicality and feasibility. Moreover, once our IRB application is approved, the neural network model will be trained and tested again by collecting training and test data sets from actual patients and applying them for left and right limb recognition in clinical upper limb orthopedic surgery. In addition, orthopedic surgery also includes lower limb orthopedic surgery. Like upper limb orthopedic surgeries, currently, lower limb orthopedic surgeries mostly use marking or barcode scanning to recognize left and right limb positions without any AI methods. Hence, we believe that the AIIRS proposed in this paper could be applied to left and right recognition in lower limb orthopedic surgery through different training data sets, test data sets, and parameter modifications of the neural network model.

We will also continue to optimize the model architecture used for training, hoping to achieve the same results with lower layers and fewer neurons (nodes), so that the system requirements are reduced and recognition speed is further improved in order to facilitate practical applications. At the moment, the system is able to run smoothly thanks to our hardware facilities.

## Figures and Tables

**Table 1:** Parameters in pipeline.config of ResNet50 [34].

Name	Value
num_classed	2
batch_size	8
fine_tune_checkpoint_type	detection

**Table 2:** Parameters in YoloV4 [35].

Name	Value
batch	64
max_batches	6000
steps	3600, 3800
width	416
height	416
classes	2
filters	21
activation function	Mish
loss function	CIOU

**Table 3:** *Confusion matrix* of AIIRS.

	*Actual Values Positive*	*Actual Values Negative*
***Predicted Positive***	***81***	***0***
***Predicted Negative***	***0***	***81***

## Data Availability

Data are contained within the article.

## References

[1] Kohn L.T., Corrigan J.M., Donaldson M.S. (2000). To Err is Human: Building a Safer Health System.

[2] Taiwan Patient Safety Notification System Annual Report. https://www.patientsafety.mohw.gov.tw/Content/Downloads/List01.aspx?SiteID=1&MmmID=621273303702500244.

[3] Susanne H., Melinda M.-G., David K.N., Aaron J.D., Isomi M.-L., Jessica M.B., Marika J.B., Jeremy N.V.M., Roberta S., Paul G.S. (2015). Wrong-Site Surgery, Retained Surgical Items, and Surgical Fires: A Systematic Review of Surgical Never Events. JAMA Surg..

[4] Mark A.P., Aaron J.B., Sean E., Alan H.D. (2013). Wrong-site Spine Surgery. J. Am. Acad. Orthop. Surg..

[5] Moshtaghi O., Haidar Y.M., Sahyouni R., Moshtaghi A., Ghavami Y., Lin H.W., Djalilian H.R. (2017). Wrong-Site Surgery in California, 2007–2014. Otolaryngol.–Head Neck Surg..

[6] Nunes C., Pádua F. (2022). A Convolutional Neural Network for Learning Local Feature Descriptors on Multispectral Images. IEEE Lat. Am. Trans..

[7] Qi Y., Guo Y., Wang Y. (2021). Image Quality Enhancement Using a Deep Neural Network for Plane Wave Medical Ultrasound Imaging. IEEE Trans. Ultrason. Ferroelectr. Freq. Control.

[8] Hassanzadeh T., Essam D., Sarker R. (2021). 2D to 3D Evolutionary Deep Convolutional Neural Networks for Medical Image Segmentation. IEEE Trans. Med. Imaging.

[9] Duan Z., Zhang T., Tan J., Luo X. (2020). Non-Local Multi-Focus Image Fusion with Recurrent Neural Networks. IEEE Access.

[10] Tian Y. (2020). Artificial Intelligence Image Recognition Method Based on Convolutional Neural Network Algorithm. IEEE Access.

[11] Tan Y., Liu M., Chen W., Wang X., Peng H., Wang Y. (2020). DeepBranch: Deep Neural Networks for Branch Point Detection in Biomedical Images. IEEE Trans. Med. Imaging.

[12] Waseem A., David M., Andrea G. (2020). Limbs Detection and Tracking of Head-Fixed Mice for Behavioral Phenotyping Using Motion Tubes and Deep Learning. IEEE Access.

[13] Zhang Y., Li S., Liu J., Fan Q., Zhou Y. (2020). A Combined Low-Rank Matrix Completion and CDBN-LSTM Based Classification Model for Lower Limb Motion Function. IEEE Access.

[14] Javeed S., Dibble C.F., Greenberg J.K., Zhang J.K., Khalifeh J.M., Park Y., Wilson T.J., Zager E.L., Faraji A.H., Mahan M.A. (2022). Upper Limb Nerve Transfer Surgery in Patients With Tetraplegia. JAMA Netw. Open.

[15] Li M., Guo J., Zhao R., Gao J.-N., Li M., Wang L.-Y. (2022). Sun-Burn Induced Upper Limb Lymphedema 11 Years Following Breast Cancer Surgery: A Case Report. World J. Clin. Cases.

[16] Shi W., Dong J., Chen J.-F., Yu H. (2022). A Meta-Analysis Showing the Quantitative Evidence Base of Perineural Nalbuphine for Wound Pain From Upper-Limb Orthopaedic Trauma Surgery. Int. Wound J..

[17] Chmelová K., Nováčková M. (2022). Effect of manual lymphatic drainage on upper limb lymphedema after surgery for breast cancer. Ceska Gynaecol..

[18] Johanna W., Carina R., Lina B.-K. (2022). Linking Prioritized Occupational Performance in Patients Undergoing Spasticity-Correcting Upper Limb Surgery to the International Classification of Functioning, Disability, and Health. Occup. Ther. Int..

[19] Ramström T., Reinholdt C., Wangdell J., Strömberg J. (2022). Functional Outcomes 6 years After Spasticity Correcting Surgery with Regimen-Specific Rehabilitation in the Upper Limb. J. Hand Surg..

[20] Duan Y., Wang G.-L., Guo X., Yang L.-L., Tian F.G. (2022). Acute Pulmonary Embolism Originating from Upper Limb Venous Thrombosis Following Breast Cancer Surgery: Two Case Reports. World J. Clin. Cases.

[21] Zhang H.-Z., Zhong Q.-L., Zhang H.-T., Luo Q.-H., Tang H.-L., Zhang L.-J. (2022). Effectiveness of Six-Step Complex Decongestive Therapy for Treating Upper Limb Lymphedema After Breast Cancer Surgery. World J. Clin. Cases.

[22] Stanley E.A., Hill B., McKenzie D.P., Chapuis P., Galea M.P., Zyl N.V. (2022). Predicting Strength Outcomes for Upper Limb Nerve Transfer Surgery in Tetraplegia. J. Hand Surg..

[23] Alsajjan H., Sidhoum N., Assaf N., Herlin C., Sinna R. (2022). The Contribution of the Late Dr. Musa Mateev to the Field of Upper Limb Surgery with the Shape-Modified Radial Forearm Flap. Ann. De Chir. Plast. Esthétique.

[24] Redemski T., Hamilton D.G., Schuler S., Liang R., Michaleff Z.A. (2022). Rehabilitation for Women Undergoing Breast Cancer Surgery: A Systematic Review and Meta-Analysis of the Effectiveness of Early, Unrestricted Exercise Programs on Upper Limb Function. Clin. Breast Cancer.

[25] Meunier V., Mares O., Gricourt Y., Simon N., Kuoyoumdjian P., Cuvillon P. (2022). Patient Satisfaction After Distal Upper Limb Surgery Under WALANT Versus Axillary Block: A Propensity-Matched Comparative Cohort Study. Hand Surg. Rehabil..

[26] Luo Q., Liu H., Deng L., Nong L., Li H., Cai Y., Zheng J. (2022). Effects of Double vs Triple Injection on Block Dynamics for Ultrasound-Guided Intertruncal Approach to the Supraclavicular Brachial Plexus Block in Patients Undergoing Upper Limb Arteriovenous Access Surgery: Study Protocol for a Double-Blinded, Randomized Controlled Trial. Trials.

[27] Manna M., Mortenson W.B., Kardeh B., Douglas S., Marks C., Krauss E.M., Berger M.J. (2022). Patient Perspectives and Self-Rated Knowledge of Nerve Transfer Surgery for Restoring Upper Limb Function in Spinal Cord Injury. PM&R.

[28] Beiranvand S., Alvani M., Sorori M.M. (2022). The Effect of Ginger on Postoperative Nausea and Vomiting Among Patients Undergoing Upper and Lower Limb Surgery: A Randomized Controlled Trial. J. PeriAnesthesia Nurs..

[29] Gao Y., Dai P., Shi L., Chen W., Bao W., He L., Tan Y. (2021). Effects of Ultrasound-Guided Brachial Plexus Block Combined with Laryngeal Mask Sevoflurane General Anesthesia on Inflammation and Stress Response in Children Undergoing Upper Limb Fracture Surgery. Minerva Pediatr..

[30] Połap D., Jaszcz A., Wawrzyniak N., Zaniewicz G. (2023). Pooling With Poisoning Detection Module for Automatic Side Scan Sonar Data Analysis. IEEE Access.

[31] Farhadi F., Barnes M.R., Sugito H.R., Sin J.M., Henderson E.R., Levy J.J. (2022). Applications of Artificial Intelligence in Orthopaedic Surgery. Front. Med. Technol..

[32] Lalehzarian S.P., Gowd A.K., Liu J.N. (2021). Machine Learning in Orthopaedic Surgery. World J. Orthop..

[33] Haeberle H.S., Helm J.M., Navarro S.M., Karnuta J.M., Schaffer J.L., Callaghan J.J., Mont M.A., Kamath A.F., Krebs V.E., Ramkumar P.N. (2019). Artificial Intelligence and Machine Learning in Lower Extremity Arthroplasty: A Review. Artif. Intell. Mach. Learn..

[34] Rashidi A., Grantner J., Abdel-Qader I., Shebrain S.A. (2022). Box-Trainer Assessment System with Real-Time Multi-Class Detection and Tracking of Laparoscopic Instruments, using CNN. Acta Polytech. Hung..

[35] Zhang X., Wang H. (2021). Research on YOLOv4 Detection Algorithm Based on Lightweight Network. Comput. Sci. Appl..

